# Most Chinese Preschool Teachers Value Guided Play Over Free Play: Latent Profiles and Associated Predictors

**DOI:** 10.3389/fpsyg.2021.780367

**Published:** 2021-11-29

**Authors:** Xunyi Lin, Yutong Liao, Manli Xue, Yeshe Colliver

**Affiliations:** ^1^College of Education, Fujian Normal University, Fuzhou, China; ^2^College of Education, Nanchang Institute of Science and Technology, Nanchang, China; ^3^School of Education, Macquarie University, Sydney, NSW, Australia

**Keywords:** play beliefs, Chinese teachers’ perspectives, early childhood education and care (ECEC), intra-cultural variation, latent profile analysis (LPA)

## Abstract

Longitudinal research suggests that optimal long-term outcomes are achieved when early childhood education and care (ECEC) balance free with guided play. A prerequisite for this achievement is that ECEC teachers value both equally. This study examines preschool teachers’ play beliefs profile and explores its association with teachers’ backgrounds (e.g., teaching experience, education level) in a sample of 674 Chinese teachers in Fujian, China. Participants completed an adapted form of the Parent Play Belief Scale, the Chinese Teacher Play Beliefs Scale (CTPBS), to report their beliefs regarding young children’s play and early academics. Latent profile analysis (LPA) revealed 91% of teachers exhibited high *Academics over Guided Play* (AGP) and low *Free Play and Socio-Emotional Skills Support* (FPSSS), whereas only 9% were high in both factors. Teachers with a decade or more teaching experience were more likely to belong to the *high AGP* and low *FPSES* profile. The findings indicate that the majority of Chinese ECEC teachers value guiding play to academic skills more than they do facilitating free play for socio-emotional skills. Professional development focused on balancing guided with free play may be necessary for the majority of Chinese ECEC teachers to catch up with the zeitgeist of contemporary international research and policy on intentional teaching in play.

## Introduction

Cross-cultural comparative studies have revealed preschool teachers from various backgrounds attribute different characteristics and significance to children’s play (e.g., Wu and Rao, 2011; van der Aalsvoort et al., 2015). For example, the emphasis on teacher-directed academic training in traditional Chinese culture has been linked to the influence of Confucianism (Zhu and Zhang, 2008). Progressive educational concepts and practices including child-centered, play-based teaching and learning have been exported from European-heritage to many Eastern countries including China since the end of the twentieth century (Rao et al., 2010; Li and Wang, 2017). It has been shown that Chinese early childhood education and care (ECEC) teachers increasingly believe that play is extremely valuable (Li and Chen, 2017). These beliefs toward play not only influence the curricula and their pedagogical practices (Parker and Neuharth-Pritchett, 2006; Yin et al., 2021), but also children’s early learning and development (Kansanen, 1991). Crucially, large longitudinal research has significantly shaped the zeitgeist of contemporary ECEC practice (Bennett, 2005; Fleer, 2010) by showing that ECEC services in which children initiate about half of their activities throughout the day lead to the best long-term outcomes (Sylva et al., 2004). Of the child-initiated activities (that is, of the “play” activities; Breathnach et al., 2017, p. 447), it was optimal for children’s long-term achievement when “almost half” were guided by adults (Sylva et al., 2004, p. 36). This means a 1:1 ratio of guided and free play is likely to be ideal (Sylva et al., 2004; Siraj-Blatchford, 2009). As a first step toward ECEC teachers reaching this ideal practice, their play beliefs must value this 1:1 ratio; as practice can only attain this balance once teachers have this intention and then gain the requisite experience in how to enact the intention (Parker and Neuharth-Pritchett, 2006; Yin et al., 2021). Yet reviews of ECEC teachers’ beliefs reveal considerable variation internationally (Bubikova-Moan et al., 2019). Few studies (e.g., Izumi-Taylor et al., 2010; Rengel, 2013; Peterson et al., 2018) have quantified ECEC teachers’ play beliefs using a validated scale, or sought to examine intra-cultural variation of beliefs that would help policy makers target interventions for changing teachers’ beliefs, and, ultimately practices to improve the quality of ECEC (Bubikova-Moan et al., 2019, p. 779). In this endeavor, from a bioecological approach (Bronfenbrenner and Morris, 2006), it is also necessary to consider the influence of socio-cultural contexts (Hedegaard, 2009) and teachers’ personal experiences (Ivrendi, 2017) on play beliefs. In particular, the broader cultural context must be understood if research endeavors to unearth intra-cultural differences in play beliefs (Haight et al., 1997). Despite powerful arguments about the significance of play for children’s learning and development (e.g., Weisberg et al., 2013a)—and play being the basis of ECEC curriculum across the developed (Organization for Economic Cooperation and Development [OECD], 2017) and, increasingly, the developing world (Gupta, 2011; Li and Wang, 2017)—quantifying the extent to which teachers’ education might influence their beliefs about play is an important first step in improving the quality of ECEC teacher education, and therefore young children’s experiences in this crucial life stage (McCoy et al., 2017). Given that teacher-guided play may be associated more with academic skills than is free play (which may be more associated with socio-emotional than academic skills) (Zosh et al., 2018), understanding whether ECEC teachers’ play beliefs mirror the 1:1 ratio of free to guided play that is indicated to be optimal for child outcomes (Sylva et al., 2004; Siraj-Blatchford, 2009) is a vital first step in raising the effectiveness of ECEC (Ulferts et al., 2019). This study, therefore, sought to quantify Chinese ECEC teachers’ play beliefs using a psychometrically sound measurement tool, the Chinese Teacher Play Beliefs Scale (CTPBS), and examine their relations to background variables such as education and experience levels.

### Play Beliefs of Early Childhood Education and Care Teachers

Play is undoubtedly an essential daily activity of early childhood and theoretically impacts children’s social, emotional, and cognitive development (e.g., Sylva et al., 2004; Weisberg et al., 2013a; Zosh et al., 2018). Yet what teachers believe about play are thought to impact if and how they implement it within their pedagogy (Pyle and Danniels, 2017; Avornyo and Baker, 2018), and therefore whether it will assist in the quality of ECEC provision (Wen et al., 2011). Play beliefs are defined broadly as tacit, often unconsciously held assumptions about play and its relation to children’s development (Haight et al., 1997; Fogle and Mendez, 2006). In extant studies, play beliefs are synonymous with values, conceptions, perceptions, perspectives, images, views, thoughts, judgments toward children’s play (Pajares, 1992; Fives and Buehl, 2012; Baustad et al., 2018; Nilsen, 2021). Adults’ beliefs contribute to positive or negative judgments about the significance of play (Fogle and Mendez, 2006). In particular, ECEC teachers’ perspectives on play are important for the enactment of the most dominant type of ECEC approaches: play-based curricula (Avornyo and Baker, 2018). Acting as a contextual “filter” (Clark and Peterson, 1986), teachers’ beliefs to some extent affect when teachers interpret, screen their classroom practices, and adapt the subsequent ones (Wen et al., 2011). Teachers’ beliefs toward play and learning influence the curricula and their pedagogical practices, as well as children’s early learning and development (Kansanen, 1991). Their beliefs are particularly important if ECEC is to have the life-long impact indicated by longitudinal research (e.g., McCoy et al., 2017), as teachers can only implement a balance of guided and free play if their values support this (Parker and Neuharth-Pritchett, 2006; Wen et al., 2011; Yin et al., 2021).

In a study by Hyvonen (2011), teachers’ play beliefs were comprised of teachers’ views on the effects of play on early childhood education, preference for the specific kinds of play, both of which determine how teachers arrange play activities. In most previous studies, the roles of teachers are varied by how teachers interact with children for better development in play activities. Some theorists argued that teacher participation may interfere with child play (e.g., Isaacs, 1929), and decades of teacher education and training have reinforced the idea that teacher involvement will only impair the maximal value of play for learning (Enz and Christie, 1993; Pyle and Danniels, 2017). Asian ECEC teachers have traditionally valued play less than their European-heritage counterparts (Bubikova-Moan et al., 2019), with beliefs in some countries (e.g., China) seeing play and learning as completely incompatible, meaning many activities—including play—are adult-guided (Wu et al., 2018). Chinese ECEC curriculum has succumbed to the influence of Western education since it began “opening up” to globalization, both in policy and practice, since the 1980s (Zhu and Zhang, 2008, p. 174), most palpably since the 1989 “Kindergarten Work Regulations and Procedures” (p. 174). This policy emphasized the value of child-initiated activity and its integration with group and other activities (Qi and Melhuish, 2017). By the 1990s, the Reggio Emilia Approach (Edwards et al., 2011), The Zone of Proximal Development (Vygotsky, 1978) and the Project Approach (Helm and Katz, 2011)—each of which acknowledge the importance of child-initiated play—were all recognized in Chinese ECEC policy (Qi and Melhuish, 2017). Such national changes sit within an international context of empirical (Siraj-Blatchford, 2009; Weisberg et al., 2013b) and policy (Bennett, 2005; Rogers, 2013) trends toward a more active teacher role in children’s play, which often calls for greater incorporation of academic content within children’s learning activities. However, these rapid reforms were challenging for Chinese teachers (Zhu and Zhang, 2008, 2018). As noted in the famous “Preschool in Three Cultures” research, the reforms’ success “depends on teachers’ understanding of how and why to teach children in the ways the Guidelines suggest” (Tobin et al., 2009, p. 85). The views of parents, who may not agree with such child-centered approaches to early learning (Zhu and Zhang, 2008; Fung and Cheng, 2012), are likely to also contribute to Chinese ECEC teachers’ current beliefs.

Other than differing beliefs about how much they value academic skills (e.g., literacy, numeracy) and child-initiated play, it is likely there is variation in beliefs how much teachers should involve themselves in children’s play, as many scholars suggest this would abilities and promote their cognitive, social, and language development (Smilansky and Shefatya, 1990; Enz and Christie, 1993; Weisberg et al., 2013b; Pyle et al., 2021). Various authors identify specific roles ECEC teachers should move between when involved in play such as stage manager, play leader, director, co-player, and uninvolved co-player (Enz and Christie, 1993; van Hoorn et al., 2014), model, mediator, allower, and afforder (Hyvonen, 2011). Indeed, the roles can be considered within the discourse of play regulation and support (Rengel, 2013), but do imply greater value on adult-determined content (e.g., academics) than a purely child-initiated approach. This might suggest that Chinese ECEC teachers have shifted from a teacher-directed curriculum to a child-centered one, only to be shifted more toward a view which values play and academics equally. Research has yet to confirm if this is so.

Previous studies have elaborated powerful arguments for the importance of play in children’s education and identified variability in roles that ECEC teachers take in child play (Fisher et al., 2013; Weisberg et al., 2013b; Zosh et al., 2018). A vital first step in ensuring teachers take the most educationally valuable role in play is to determine the beliefs underpinning their practices. Given the culturally situated nature of these beliefs (Bronfenbrenner and Morris, 2006), a key piece in this first step is understanding the cultural beliefs of the most populous country in the world. China has received profound influence from Confucian heritage culture (CHC), which values academic training in early childhood and highlights traditional teaching approach on children’s development (Zhu and Zhang, 2008). In this cultural context, Chinese teachers may be regarded as an educational authority whose direction has long been widely encouraged, meaning the value of child-initiated, play-based activity has been overlooked (Li, 2012). Coupled with the lack of “faith” that many parents have in play, it is likely that there is some diversity in teachers’ beliefs, as has been found in qualitative work in Chinese heritage (e.g., Fung and Cheng, 2012, p. 24) and European heritage contexts (e.g., Pyle and Danniels, 2017; Colliver, 2019). However, limited studies have examined the diversity in Chinese ECEC teachers’ beliefs toward children’s play. This study, therefore, aims to explore the variation in Chinese ECEC teachers’ play beliefs.

### Person-Centered Study of Play Beliefs Within a Cultural-Historical Framework

To examine the intra- cultural variation of Chinese ECE teachers’ beliefs, we situated the study within a cultural-historical theory methodological framework, which aims to provide a “holistic” view of the phenomenon studied (Chaiklin, 2012, p. 209). For Rogoff (2003) “the interpersonal, personal, and cultural-institutional aspects of the event constitute the activity… [n]o aspect exists or can be studied in isolation from the others” (p. 58). For the purposes of the current research questions, this approach is about seeing teachers’ beliefs not as isolated from their context, but rather informing and informed by the larger societal, cultural, historical spheres, as well as the sub-cultures of each ECE setting (or preschool), the children attending, and their families (Bronfenbrenner and Morris, 2006). To do so, cultural-historical research examines activity and motive in one sphere as its units of analysis, maintaining the others “in the background” (Matusov, 2007, p. 324). Vygotsky, the grandfather of cultural-historical theory, saw this process as akin to studying the molecules within a drop of water to make inferences about the ocean; a “form of analysis (fundamental to) development of theories of thinking …relies on the partitioning of the complex whole into units… those units in which the characteristics of the whole are present” (Vygotsky, 2004, p. 37). To Rogoff’s (1995) three main planes of analysis—the individual, interpersonal, and cultural-institutional—Hedegaard (2008, p. 46) added the *societal* as a broader plane again. For the purposes of investigating intra-cultural variation in teachers’ beliefs, it is appropriate to examine teachers’ beliefs at the “institutional” level, as the beliefs are tied to the ECEC institution as their workplace (Colliver, 2019). In keeping with the focus on beliefs, it is possible to focus on participants’ values within a cultural-historical approach, as they represent motives at the institutional level (Hedegaard, 2009), and motives are the key unit of analysis in cultural-historical research (Fleer, 2008). Values are often considered to be part of teachers’ play beliefs (Nilsen, 2021).

Akin to these four levels of analysis, psychometric research can be conducted using variable-centered, person-centered, and person-specific approaches (Howard and Hoffman, 2018). Just as an institutional-level analysis will examine variation within a subcultural group of society (e.g., Chinese ECE teachers), despite its misleading name, a person-centered approach will determine if subgroups exist within a given population (Howard and Hoffman, 2018). This approach contrasts societal-level psychometric research, which most often uses variable-centered approaches to examine homogeneous groups of observations based on the combination of some independent variables (Bubikova-Moan et al., 2019). Variable-centered approaches may also examine the relationship of variables with play-learning beliefs (Mooi and Sarstedt, 2011; Avornyo and Baker, 2018). Notwithstanding, variable-centered analyses tend to ignore the intra-population variability, for they assume that associations are similar across all the participants (Howard and Hoffman, 2018). For example, Avornyo and Baker (2018) investigated Ghanaian early years stakeholders’ play beliefs along the single dimension (i.e., the role of play in children’s learning), roughly categorizing their play beliefs according to levels of education, and examined whether their education status could predict play-learning beliefs individually. However, it was noted that components of teachers’ beliefs systems are often not isomorphic, and thus there exists the potential for belief variables to combine in novel ways that shape outcomes. Finally, person-specific approaches are used to examine relationships between variables at the individual level, and are inappropriately fine-grained for research on Chinese ECEC teachers’ beliefs.

In contrast with studying ECEC teachers’ play beliefs in isolation, person-centered approaches allow unique arrangements of play beliefs to be examined in association with their predictors or outcomes. Unlike variable-orientated procedures, the person-centered methodology can look at multiple ideas of addressing a specific issue, and better analyze multidimensional and correlated data (Bámaca-Colbert and Gayles, 2010). ECEC teachers with different cultural backgrounds emphasize different characteristics and significance of children’s play (Sandberg and Samuelsson, 2003; Wu and Rao, 2011; van der Aalsvoort et al., 2015). In addition to culture, childhood experience is also considered key to beliefs and perceptions that are associated with adult beliefs and behavior (Thompson et al., 2008). Prior studies have reported variability in roles that teachers enact in children’s play, as well as fluctuations in the role types (Johnson et al., 1999, p. 201; Pyle and Danniels, 2017). A more nuanced understanding could be garnered about the types of play beliefs among sub-populations of individuals identified based on within-group similarities and between-group differences in associations among variables. Person-centered approaches to analysis are well positioned to provide such an understanding (Howard and Hoffman, 2018).

To overcome apparent disadvantages of variable-centered analysis (Li and Chen, 2017; Yang and Li, 2018; Lin et al., 2020), this study applied a person-centered approach *via* latent profile analysis (LPA) to explore the variation in ECEC teachers’ play beliefs.

#### Play Beliefs of Early Childhood Education and Care Teachers With Different Backgrounds

Because the ECEC teacher holds a (if not *the most*) crucial role in the early education system, it is paramount that any investigation of their personal conceptualization and acceptance of the role is holistic (Edwards et al., 2011). Thus, concerning teachers’ play beliefs, it is necessary to consider the influence of personal characteristics and socio-cultural contexts, from individual to society. Ivrendi’s (2017) study showed that experienced teachers were busy with tasks unrelated to children’s play more often than less experienced teachers. In addition, cross-culturally comparative studies have indicated that ECEC teachers with different cultural backgrounds emphasize different characteristics and significance of children’s play (Izumi-Taylor et al., 2010; van der Aalsvoort et al., 2015). For example, none of the Japanese ECEC teachers in Izumi-Taylor et al. (2010) linked play to academic learning but teachers in the United Kingdom, United States, Germany, and Sweden did. Such differences across contexts might stem from variations in traditional cultural conceptions of how children develop (Avornyo and Baker, 2018). It is noted that, apart from differences, there exist some similarities in perceptions on play beliefs among early childhood educators from different backgrounds. Since progressive educational ideas and practices have been imported from European-heritage countries to other countries including China, more Chinese education authorities and parents have increasingly believed that play is a valuable medium for children’s learning and development (Jiang and Han, 2016; Li and Chen, 2017).

Research focused on cross-cultural variation in ECEC teachers’ beliefs on children’s play has detracted from examination of intra-cultural variation in extant studies (Izumi-Taylor et al., 2010; Rengel, 2013; Peterson et al., 2018). For this reason, the current study draws on a person-centered approach to focus on the diversity in ECEC teachers’ beliefs toward children’s play in China and examine its relations to their backgrounds.

### The Current Study

Acknowledging the diversity and complexity of prior investigations of ECEC teachers’ play belief**s**, the current study uses a person-centered approach *via* LPA to identify various profiles of individuals based on their play beliefs. Theoretically, we intend to contribute to the field through empirical evidence and substantiating the person-centered approach in play belief research. Researchers can acquire a more parsimonious but nuanced understanding of the play beliefs of different backgrounds. Practically, it informs early education authorities and practitioners of a convenient way to identify patterns of teachers’ play beliefs and provide them with differentiated support.

Overall, the present study aims to: (a) identify patterns of ECEC teachers’ play beliefs; (b) examine how these patterns are related to teachers’ backgrounds; The following research questions guided this investigation:

(1) What group profiles of play beliefs would emerge from Chinese ECEC teachers?
(2) How are these profiles associated with teachers’ background variables?

## Materials and Methods

### Participants

This study was conducted in Fuzhou, the capital city of Fujian province, located in southeast China. Participants were informed that they could withdraw participation anytime and informed consent was given at the start of the survey. A total of 674 ECEC teachers were recruited from a variety of kindergartens, in both urban and rural areas to ensure sample diversity. As shown in Table 1, 172 teachers (25.5%) had more than 10 years of teaching experience. The majority (65.4%) of the teachers had an associated or lower degree and 34.6% of the teachers had a bachelor or above degree. Almost all (90.1%) teachers specialized in preschool education. Around half (49.7%) had no teaching qualification.

**TABLE 1:** Participant characteristics (*N* = 674).

Demographic characteristics	Frequency (%)
**Education level**	
High school and below	89 (13.2)
Associated degree	352 (52.2)
Bachelor degree and above	233 (34.6)
**Teaching qualification**	
Yes	339 (50.3)
No	335 (49.7)
**Degree education major**	
Preschool education	607 (90.1)
Others	67 (9.9)
**Years teaching experience**	
0–3 Years	250 (37.1)
4–9 Years	252 (37.4)
10 Years and above	172 (25.5)

### Measures

#### Demographic Questionnaire

In the questionnaire format, teachers reported their years teaching experience, education major, education level, and teaching qualification. Teachers were also required to provide basic information about their kindergartens, such as the location (urban or rural) and type (public or private).

#### Chinese Teacher Play Beliefs Scale

**FIGURE 1:**
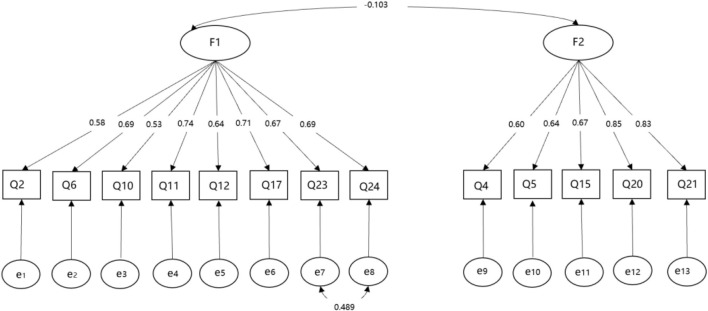
Confirmatory factor analysis of ECEC teacher play beliefs.

The CTPBS was developed and validated to assess Chinese ECEC teachers’ beliefs toward young children’s play and learning. Existing scales were mostly designed for parents and had not been adapted for ECEC teachers in China. In this study, we adapted the current parental play belief scale which used play vs. academics value dimension (e.g., Fogle and Mendez, 2006; Lin and Li, 2018), to include a child- vs. adult-guided play dimension, consistent with the international trends toward greater guidance of play by adults (Bennett, 2005; Colliver, 2019) and evidence that a balance between guided and free play is likely to be optimal for children’s long-term achievement outcomes (Sylva et al., 2004; Siraj-Blatchford, 2009). Scale development processes resulted in a 13-item CTPBS scale (Watson et al., 1988). These included item assessment by experts in child development and play to confirm the scale’s content validity, and cross-cultural adaptation considerations for use with self-report data, including translating the English version to Mandarin, then back-translating these Mandarin versions to English again by a translator who was blinded to the original English version (Beaton et al., 2000; see Table 2). The factor structure of the scale was explored through principal component analyses with an Orthogonal (varimax) rotation method. Some principal criteria were used as a guide: (1) each retained factor has an eigenvalue larger than one; (2) the factor solutions explain substantial proportion of the total variance with each factor accounting for more than 10% of the total variance; (3) item communality values were 0.40 or greater; (4) the item should have no cross-loadings among factors (5) each factor has at least three items and (6) factors demonstrate meaningfulness and interpretability in the content of their items. A two-factor model was selected for its best meeting to all criteria described above (see Table 2). CTPBS could be summarized by two factors, accounting for 55.86% of the total variance (32 and 23%, respectively). As shown in Figure 1, a confirmatory factor analysis (using maximum likelihood extraction) for the two-factor model indicated that the model fit was satisfactory (χ^2^ = 246.376, *DF* = 63, *P* = 0.000, CFI = 0.947, TLI = 0.935, RMSEA = 0.076, SRMR = 0.053).

**TABLE 2:** Structure and loadings of CTPBS for principal component analysis with varimax/promax rotation.

Scale item/version translated back from mandarin	Factor 1	Factor 2
**Academics over guided play (AGP) (alpha = 0.86)**		

Q2. Child-initiated free play does not help children to learn academic skills like counting or recognizing letters/Free play initiated by children does not help them learn academic skills like counting or word recognition	**0.631**	–0.05
Q6. It would be more beneficial to read to children than to join in on their play/Reading to children is more beneficial than participating in his/her play.	**0.713**	0.014
Q10. My child will get more out of play if I join in/Children will get more out of free play if I join in the play.	**0.561**	0.28
Q11. It is more important for children to have good academic skills than to play well with others/It is more important for children to have good academic skills than to play well with others.	**0.754**	–0.019
Q12. Free playtime is not an everyday high priority in my class/Free playtime is not a priority for me every day in my class/kindergarten.	**0.716**	–0.159
Q17. I do not think children learn important skills through play without adult guidance/I do not think children can learn important skills thorough play without adult guidance.	**0.757**	–0.052
Q23. I should structure and guide about half of children’s play/Half of the children’s play should be structured and directed by me.	**0.767**	–0.045
Q24. Play is best when adults structure it, to maximize learning/Play structured by adults is best for children to maximize learning.	**0.799**	–0.012

**Free Play and Socio-Emotional Skills Support (FPSSS) (alpha = 0.84)**		

Q4. I have a lot of fun with children when we play together/I have a lot of fun with children when we play together.	–0.054	**0.647**
Q5. Free play can improve children’s language and communication abilities/Free play can improve children’s language and communication skills.	–0.054	**0.691**
Q15. Free play can help children to learn to express his or her feelings/Free play can help children learn to express his/her feelings.	0.09	**0.775**
Q20. Through free play, children develop new skills and abilities/Children can develop new skills and abilities through free play.	–0.02	**0.874**
Q21. Children gain social skills during free play/Children learn social skills through free play.	–0.062	**0.861**

*Note: Salient Factor Loadings are bolded.*

In the CTPBS, teachers are required to complete a 5-point Likert scale (1 = extremely disagree to 5 = extremely agree) to evaluate their beliefs regarding young children’s play and learning. The academic focus subscale included eight items that highlight the acquisition of knowledge and skills rather than free play for young children. For example, “Free play initiated by young children does not help them learn intellectual skills like counting or word recognition.” The play support subscale contained 5 items that reflect an attention to the importance of child-free play, such as “Free play can improve children’s language and communication skills.” Cronbach’s alpha coefficient was 0.79 for the CTPBS scale. For the two subscales, Cronbach’s alphas coefficients were 0.86 and 0.84, respectively.

### Theoretical Framework and Statistical Analyses

The cultural influences nature on teachers’ beliefs makes cultural-historical theoretical framework most appropriate for the current investigative focus (Haight et al., 1997; Hedegaard, 2008). Within the tiered structure of influences of activity (societal, institutional, interpersonal and individual levels; Rogoff, 1995; Hedegaard, 2008), the institutionally nested nature of Chinese ECEC teachers’ beliefs determined our institutional-level focus on values and beliefs, as discussed earlier. This level of analysis fits with the person-centered approach described within Howard and Hoffman’s (2018) three levels of approach to psychological research, which, it is argued, should be chosen for its fit with the research question(s).

In the present study, LPA was used to discover diverse patterns of teachers’ play beliefs. As a person-centered statistical approach, LPA allows researchers to find previously unnoticed subgroups among varied populations (Vermunt and Magidson, 2002; Vermunt, 2010). In comparison to traditional cluster analysis, LPA uses rigorous model-fit indices to determine the number and character of the profiles, resulting in higher sensitivity to latent taxonomy detection (Cleland et al., 2000). To get the proper latent profile solution, we employed the following commonly used model-fit indices (Morin et al., 2011; Foti et al., 2012): The Akaike Information Criterion (AIC), Bayesian Information Criterion (BIC), and sample-size adjusted BIC (SSA-BIC), which should be smaller than those of other profiles; the Entropy value should be higher; the Vuong-Lo-Mendell-Rubin likelihood ratio statistics (VLMR) value and Bootstrap Likelihood Ratio Test (BLRT) value should be significant (*p* < 0.05). In addition to the above fitting indices, the solution of the model should also consider the theoretical limitations.

First, the mean scores of the two CTPBS subscales “Academics over Guided Play” (AGP) and “Free Play and Socio-Emotional Skills Support” (FPSSS) were employed as indicators to identify the latent profiles of teachers’ play beliefs. Next, a series of LPA models were conducted in Mplus Version 8.0 using the full information maximum likelihood (FIML) as the estimation method (Muthén and Muthén, 1998–2010). Then, teachers’ background information variables were used as predictors of the latent profile variable. Logistic regression was conducted in Mplus Version 8.0 with the indicators of years teaching experience, education major, education level, and teaching qualification to evaluate how these factors were associated with the Chinese ECEC teachers’ play beliefs patterns.

## Results

The means, standard deviations and intercorrelations of variables are presented in Table 3. Play Support was positively related to teachers’ education level (*r* = 0.192, *p* < 0.001), years teaching experience (*r* = 0.138, *p* < 0.001), and teaching qualification (*r* = 0.110, *p* < 0.01). Academic Focus was positively related to teachers’ education major (*r* = 0.109, *p* < 0.01), whereas negatively related to years teaching experience (*r* = –0.151, *p* < 0.001), education level (*r* = –0.194, *p* < 0.001), and teaching qualification (*r* = –0.185, *p* < 0.001) of ECEC teacher. Teachers’ years teaching experience was positively related to their teaching qualification (*r* = 0.119, *p* < 0.01) and education level (*r* = 0.135, *p* < 0.001).

**TABLE 3:** Means, standard deviations and intercorrelations of variables in this study.

	*M* (*SD*)	1	2	3	4	5	6
1. Education level		1					
2. Teaching qualification		0.542***	1				
3. Degree education major		–0.229***	–0.161***	1			
4. Years teaching experience		0.135***	0.119**	–0.103**	1		
5. AGP	4.53 (0.84)	0.192***	0.110**	–0.075	0.138***	1	
6. FPSSS	2.61 (0.49)	–0.194***	–0.185***	0.109**	–0.151***	–0.083*	1

*p < 0.05, **p < 0.01, ***p < 0.001.

*AGP, Academics over Guided Play; FPSSS, Free Play and Socio-Emotional Skills Support.*

### Latent Profile Analysis of Teachers’ Play Beliefs

The first research question in this study examined Chinese ECEC teachers’ play beliefs profiles. Latent profile models ranging from 1 to 5 classes were estimated. As shown in Table 4, a two-profile model was adopted as it had the highest entropy (0.90), and a significant VLMR and BLRT value (*p* < 0.001). Despite having the lowest AIC, BIC, and SSA-BIC value, the four-profile structure’s VLMR value was insignificant. In consideration of the model indices and the interpretability of the profiles, a two-profile solution was adopted as the final model in the present study.

**TABLE 4:** Latent profile analysis for Teacher’s play beliefs.

	1	2	3	4	5
AIC	2636.310	**2528.216**	2470.826	2270.953	2276.953
BIC	2654.363	**2559.809**	2452.958	2329.625	2349.164
Sample-size adjusted BIC	2641.663	**2537.583**	2421.208	2288.348	2298.363
Entropy		**0.900**	0.790	0.886	0.876
VLMR *p*-value		**0.0000**	0.5118	0.6768	0.7448
BLRT *p*-value		**0.0000**	0.0000	0.0000	1.0000
%Profile1	100%	**91.1%**	27.0%	3.1%	3.1%
%Profile2		**9.0%**	66.2%	55.2%	55.2%
%Profile3			6.8%	6.7%	0.0%
%Profile4				35.0%	35.0%
%Profile5					6.7%

*n = 674. Note: Bolded values signify best fit.*

**FIGURE 2:**
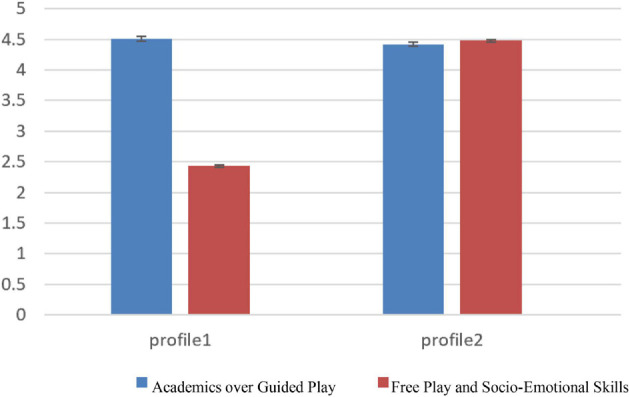
Latent profile analysis results for Teacher’s play beliefs.

As shown in Figure 2, Profile 1 was distinguished by a high level of *AGP* (*M* = 4.51, *SD* = 0.04) and a low level of *FPSSS* (*M* = 2.43, *SD* = 0.02). Profile 2 was distinguished by high scores on both *AGP* (*M* = 4.42, *SD* = 0.03) and *FPSSS* (*M* = 4.48, *SD* = 0.02). The LPA results identified two profiles: (1) *High AGP and Low FPSSS* (91.1%), (2) *High AGP and FPSSS* (9.0%).

### Factors Associated With Teachers’ Play Beliefs Profile

The second research question of this study was to identify whether teachers’ years teaching experience, education major, education level, and teaching qualification predicated Chinese ECEC teachers’ play beliefs profile. Binary logistic regression was conducted in Mplus using the above indicators to evaluate how these factors related to the likelihood of membership in a Chinese ECEC teachers’ play beliefs profile.

As shown in Table 5, comparing the *High AGP and Low FPSSS* profile (Profile 1) to the *High AGP and High FPSSS* profile (Profile 2), there was a significant difference in years teaching experience. Teachers with 10 years and above of teaching experience were more likely to belong to latent profile 1 (Logits = 1.624, *p* < 0.01, OR = 5.257) than those with 0–3years of teaching experience. There were no significant differences (*p* > 0.05) in teachers’ education major, education level, and teaching qualification comparing Profile 1 to Profile 2.

**TABLE 5:** Binary logistic regression results of Teacher’s play belief profiles.

Reference group	Comparison group			
	Profile 1			
	
Profile 2	Predictor	Logits	S.E.	O.R.
	Teaching experience			
	0–3 years	–	–	–
	4–9 years	0.525	0.300	1.731
	10 years and above	1.624**	0.495	5.257
	Major			
	Preschool education	–	–	–
	Others	0.035	0.484	1.036
	Education			
	High school and below	–	–	–
	Associated degree	–0.453	0.507	0.636
	Bachelor degree and above	–0.271	0.595	0.763
	Teacher qualification			
	No	–	–	–
	Yes	0.056	0.336	1.058

**p < 0.01, n = 674.

## Discussion

### Constructs and Patterns of Chinese Early Childhood Education and Care Teachers’ Play Beliefs

In collaboration with ECEC teachers and experts in China, this study developed, revised, and validated the *Chinese Teachers’ Play Beliefs Scale* (CTPBS). First, a 27-item pilot version of the measure was culturally adapted by the final author based on the current parental play belief scale (Fogle and Mendez, 2006), then the first author translated it from English into Chinese. A group of Chinese ECEC teachers were asked to comment on the scale’s wording and suitability and ECEC experts were consulted with to confirm the scale’s content validity. In the process of EFA, 14 items were removed because of unacceptably low communities, their lack of significant loadings on the two factors, or cross-loading among factors. The final version of CTPBS was a 13-item scale, which included two constructs: “Academics over Guided Play” (AGP) and “Free Play and Socio-Emotional Skills Support” (FPSSS), which had similar factors of parental play belief scale to Fogle and Mendez (2006) study. However, in line with recent international trends toward intentional teaching in play (Bennett, 2005; Siraj-Blatchford, 2009), the CTPBS further explored not only attitudes on the benefits of child-centered free play, but also adult’s participation in child play, and the corresponding focus on skills learned from free and guided play (see Zosh et al., 2018). The items of the CTPBS also seemed to be more generalized than those in the Chinese Parent Play Beliefs Scale (CPPBS; Lin and Li, 2018), which was derived from a detailed list of young children’s play and preacademic activities. While CTPBS may share some concepts similar to CPPBS—play for fun and play for learning, their differences probably exist because concrete items were appropriate for teachers (as professionals intimately familiar with this nomenclature) but not for parents.

Using a person-centered approach *via* LPA, the present study revealed a two-profile model: Profile 1, who placed the highest value academic learning (e.g., counting, recognizing letters, reading), a slightly lower value on guided play, and an even lower value on free play and socio-emotional skills (e.g., language, communication, feeling expression, social skills); and Profile 2, who placed a high value both on academic and socio-emotional learning, as well as on guided and free play, albeit to a lesser extent than on the learning. Chinese parents may value adult-guided activities over free play because they believe adult-guided activities are more effective for children’s academic learning, which they value more (Rao and Li, 2009; Fung and Cheng, 2012; Sun and Rao, 2017). Interestingly, the item –“It would be more beneficial to read to children than to join in on their play”—which loaded well (0.713) onto the first subscale, AGP, suggested that adult-led instruction such as reading was valued more than even guided play, similar to this value hierarchy shown by many Chinese parents (Fung and Cheng, 2012; Sun and Rao, 2017). Similarly, Q11, “It is more important for children to have good academic skills than to play well with others” suggests a valuing of academics over play that provides a nuance to the valuing of guided play that may be quite culturally specific to Asia, as suggested in the systematic review of Bubikova-Moan et al. (2019). Like profiles of Chinese parental play beliefs where parental involvement moderated children’s academic-related play only if those parents valued play for learning, and not if they valued play just for fun (Lin and Li, 2018; Lin et al., 2019), profiles in the current study showed distinct intracultural groups of ECEC teachers who value free play differently. In contrast to the findings of prior studies, the majority of ECEC teachers valued their role in play for children’s academic learning above the value of free play for child-initiated learning of socio-emotional skills (Chakravarthi, 2009; Rengel, 2013; Clevenger, 2016; Avornyo and Baker, 2018). Chinese ECEC teachers’ valuing of their active role in play contrasts patterns found in western-heritage countries, where teachers are reluctant to “hijack” play’s inherent value (e.g., Pyle and Danniels, 2017, p. 274; Colliver, 2019). Similarly, Turkish teachers with the most experience (> 15 years) were most likely to be uninvolved in play (Ivrendi, 2017). The majority of Chinese ECEC teachers still appear to value academic learning—and to a lesser extent, guided play—over free play and socio-emotional skills. However, those with less experience, most likely newer teachers, valued guided and free play equally, suggesting they have “caught up” with contemporary trends toward valuing intentional teaching and guided play (Bennett, 2005; Siraj-Blatchford, 2009; Weisberg et al., 2013b), and possibly the contemporary emphasis on valuing and enacting both guided and free play equally (Sylva et al., 2004; Zosh et al., 2018). However, the challenge for Chinese policy is to ensure this valuing of free play and child autonomy is enacted well, as research suggests that it takes years of experience to know how to convert these beliefs to practice (Parker and Neuharth-Pritchett, 2006; Wen et al., 2011; Yin et al., 2021). While there is much rhetoric regarding the moral imperative to uphold children’s human right to play (Colliver and Doel-Mackaway, 2021), it requires skill and expertise to follow children’s interests and initiatives in a thoughtful and educational manner (Edwards et al., 2011; Hedges and Cooper, 2016).

In China, the central status of play as the principal activity in kindergarten was enshrined in the 2001 *Guidelines for Kindergarten Education Practice* (The Ministry of Education of the People’s Republic of China, 2001). With educators responding positively to the policies, great changes have taken place in kindergartens across China. Anji play, a successful experiment in child-initiated outdoor play at preschool, is being expanded across the country and learned by ECEC educators throughout the world (Wang, 2018). The current results are consistent with the idea that the benefits of a child-centered, play-based curriculum have more recently become widely accepted by Chinese ECEC teachers. Thus, Profile 2 in this study unsurprisingly valued guided and free play equally. Many preschool educators, however, may be influenced to the expectations of the families that they serve to nurture early learners and prepare young children for later academic learning (Fung and Cheng, 2012; Sun and Rao, 2017), and this tendency appears to be reflected particularly in Profile 1, high in AGP, academic learning over guided play support, and low in FPSSS. Moreover, since the importation of notions and practices imported from European-heritage countries, there has exited a conflict and tension between “host” traditional values and “guest” ones, where adults adopt different patterns of acculturation and adjustment of their culturally based beliefs about play (Berry, 1997; Rao et al., 2010). Confucian heritage culture appears to deeply rooted in China, with some teachers still tending to view recreation as akin to laziness, and playing as not being useful, with high expectations of children’s academic achievement (Lee, 1996; Li, 2003; Rao and Chan, 2009). If the 1:1 ratio of adult- and child-initiated activities, and 1:1 ratio of adult- and child-guided play (Sylva et al., 2004), is also optimal in the Chinese context, these background influences remain a significant challenge in the enterprise of improving Chinese ECEC provision: for more experienced teachers because they hold views less consistent with contemporary research evidence, and for less experienced teachers because they are more likely to hold less managerial and therefore influential roles.

### Teachers’ Backgrounds Associated With Play Belief Patterns

Studies have suggested that teachers’ educational background and teaching experience are related to teaching beliefs (Spodek, 1988; Charlesworth et al., 1993; Calderhead, 1996). The current study found that years of teaching experience predicted the profiles of ECEC teachers’ play beliefs. However, in contrast with those with less teaching experience, teachers with a decade or more teaching experience were less likely to have a free play and socio-emotional skill focus. There were no significant differences in teachers’ education major, education level, and teaching qualification comparing Profile 1 to Profile 2.

These findings contrast those of previous studies, which have suggested that more experienced Chinese teachers have more positive views on the protection of children’s right to play and an endorsement of child-centered free play (Liu, 2014). Since child play is regarded as the principal activity in ECEC, how to better support and guide child play has become the fundamental component and focus of teacher training and professional learning. On the other hand, previous studies (e.g., Wu et al., 2018) have generally shown Chinese ECEC teachers have valued play less than those from Western-heritage countries (as highlighted by Q6 and Q12 of the AGP), with the growing influence of western ideas, it seems likely that more recently trained teachers would espouse values more supportive of free play and holistic learning. It is possible that the current study is showing newer teachers have had more contemporary training reflecting an equal emphasis on guided and free play, and holistic learning as per the balancing of academic and socio-emotional learning. It is notable that previous research has shown that more teaching experience can be a predictor of higher teacher self-efficacy, which is related to the autonomy of teachers’ professional development (Cheung, 2008; von Suchodoletz et al., 2018) as well as their likelihood of enacting a play-based curriculum, which may involve minimal adult intervention in play (Yin et al., 2021). On the other hand, Chinese teachers with less teaching experience may be more likely to enact the recent ECEC education reforms emphasizing play-based, individualized and active learning (Liu and Feng, 2005). However, implementing these values in practice may require experience these teachers do not yet possess (Parker and Neuharth-Pritchett, 2006; Yin et al., 2021), or specialist in-service training (Wen et al., 2011; Hedges and Cooper, 2016; Colliver, 2019).

The changing values of Chinese ECEC teachers suggested by the current data are likely reflective of the rising influences of the Zone of Proximal Development (Vygotsky, 1978) and Reggio Emilia (Edwards et al., 2011) and Project (Helm and Katz, 2011) approaches in China in the late twentieth and early twenty-first centuries (Qi and Melhuish, 2017), the relative value of free play was significantly lower for the group who received teacher training more than 10 years ago (although such approaches can be effectively used to balance child- and teacher-oriented activities; Veraksa et al., 2021). A review of the prominent ECEC literature published in Mandarin over the past two decades reflects a redirection of value orientation in ECEC teachers’ beliefs about free play and learning socio-emotional skills. Affected by Western ECEC concepts and the Developmentally Appropriate Practice (DAP) approach (McMullen et al., 2005), Chinese literature in the early twenty-first century paid more attention to the value of child-centered play, children’s autonomy and holistic learning, including an emphasis on social and emotional skills (e.g., Qiu, 2001; Jing and Chen, 2002). However, partly due to parental expectations (e.g., Sun and Rao, 2017) and some empirical evidence (e.g., Hu et al., 2017), there has been a recent redirection in the play literature toward striking a balance between child-initiated and teacher-led play, with more emphasis on teachers’ active role in supporting and guiding play for learning (e.g., Lin et al., 2019; Deng, 2021). It is possible that this change of values, and potentially from their influence on teacher education, is reflected in the significant belief differences found here between teachers with more or less experience.

### Limitations and Future Research Directions

Several limitations need to be noted when interpreting the current findings and addressed in future studies. First, the sample was limited to teachers in Fuzhou, a highly developed city in China. There is a need for future studies to involve ECEC teachers from less-developed areas of China, to examine play beliefs more comprehensively in contemporary China. Second, some researchers have argued that the improvement of the statistical ability of LPA would be brought from more class indicators and a larger sample size (Nylund et al., 2007). Therefore, it is desirable that further studies would be conducted with larger samples using more class indicators. Third, the data about play beliefs were reported by ECEC teachers, which may lead to self-report bias or socially desirable response sets. In further research, apart from teachers’ self-reports, researchers might consider using other-informant ratings or observation. Fourth, previous studies have investigated parental and teachers’ play beliefs, but few have examined whether ECEC administrators and teachers differ in how they view child play, which is needed to be explored in future studies. Fourth, although the Parent Play Belief Scale was here adapted to reflect contemporary international trends toward balancing guided and free play (Bennett, 2005; Siraj-Blatchford, 2009), many of the items did not meet communality criteria outlined in the section “Materials and Methods”. This resulted in a smaller scale than would be anticipated if using factors derived from the original scale (Fogle and Mendez, 2006). Finally, our study did not explore and verify the cause-effect relationship between teachers’ play beliefs and child outcomes, which calls for further studies considering these variables (e.g., Lin and Li, 2018).

## Conclusion

This study suggested that Chinese ECEC teachers with more teaching experience value free play and socio-emotional learning less than their less experienced counterparts. A key contribution of this research is that it offers a data-driven typology of Chinese ECEC teacher groups based on their unique configuration of play beliefs, confirming patterns elucidated in prior, predominantly qualitative research (Rao and Li, 2009; Bubikova-Moan et al., 2019) and quantifying them. Moreover, using person-centered methodology *via* LPA grounded in a cultural-historical framework, this study explored the profiles of Chinese teachers’ play beliefs and the associations with their backgrounds, which extended prior studies that have typically relied on variable-centered approaches to examine ECEC teachers’ play beliefs. The results of this study, although preliminary, have implications for teacher training, theory and policy. The extant literature suggests that there exist tensions not only between Chinese traditional and practices and Western-heritage beliefs but also between the play-based values orientation of educators and academic-skills focus of parents in contemporary China (Sun and Rao, 2017). Limited empirical research, however, has explored the play beliefs of ECEC teachers within the context of the internal and external tensions and changes in the value of play in China. This study has begun to contribute to knowledge about the status of play in Chinese ECEC, indicating not just the valuing guided over free play and academic over socio-emotional skills, but also the variability within a minority (∼9%) of Chinese ECEC teachers’ attitudes showing greater valuing of socio-emotional learning *via* free play. If a balance of guided and free play is optimal in the Chinese context, as it has shown to be in Western contexts (e.g., Sylva et al., 2004), the current relationship between the two patterns of play beliefs and their teaching experience suggests that in-service training resources could be focused on helping older teachers to balance the academic-oriented and child-centered play activities at preschool, especially how that might be achieved using free play that allows child-led learning and autonomy. At the policy level, the existing profiles of Chinese teachers suggest that educational authorities and kindergarten principals in China might seek to train more experienced teachers in the ideal balance between guided and free play (Sylva et al., 2004; Siraj-Blatchford, 2009), and less experienced teachers in how to implement free play in the presumably more nuanced ways seen by in the practice of their more experienced counterparts (Parker and Neuharth-Pritchett, 2006; Wen et al., 2011). The current findings, therefore, imply that there remains some work for Chinese ECEC practice and policy to meet the recommendations implicit in the evidence base on balancing guided and free play and the international push for intentional ECEC teachers who value and implement adult- and child-initiated activities equally.

## Data Availability Statement

The raw data supporting the conclusions of this article will be made available by the authors, without undue reservation.

## Ethics Statement

Ethical review and approval was not required for the study on human participants in accordance with the local legislation and institutional requirements. The patients/participants provided their written informed consent to participate in this study.

## Author Contributions

XL co-designed the survey instrument, conducted the statistical analyses, and drafted the manuscript. YL and MX assisted with data collection, extraction and analysis, and also drafted the introduction and literature review. YC determined the research questions and focus, co-designed the survey instrument, and edited the manuscript. All authors contributed to the article and approved the submitted version.

## Conflict of Interest

The authors declare that the research was conducted in the absence of any commercial or financial relationships that could be construed as a potential conflict of interest.

## Publisher’s Note

All claims expressed in this article are solely those of the authors and do not necessarily represent those of their affiliated organizations, or those of the publisher, the editors and the reviewers. Any product that may be evaluated in this article, or claim that may be made by its manufacturer, is not guaranteed or endorsed by the publisher.
